# Characterization of Natural Killer Cell Profile in a Cohort of Infected Pregnant Women and Their Babies and Its Relation to CMV Transmission

**DOI:** 10.3390/v16050780

**Published:** 2024-05-14

**Authors:** Chiara Pighi, Arianna Rotili, Maia De Luca, Sara Chiurchiù, Francesca Ippolita Calò Carducci, Chiara Rossetti, Loredana Cifaldi, Roberto Bei, Leonardo Caforio, Stefania Bernardi, Paolo Palma, Donato Amodio

**Affiliations:** 1 Research Unit of Clinical Immunology and Vaccinology, Bambino Gesù Children’s Hospital, IRCCS, 00165 Rome, Italy; chiara.pighi@opbg.net (C.P.); arianna.rotili@students.uniroma2.eu (A.R.); chiara.rossetti@opbg.net (C.R.); paolo.palma@opbg.net (P.P.); 2 PhD Program in “Immunology, Molecular Medicine and Applied Biotechnologies”, Department of Systems Medicine, University of Rome “Tor Vergata”, 00133 Rome, Italy; 3 Infectious Disease Unit, Bambino Gesù Children’s Hospital, IRCCS, 00165 Rome, Italy; maia.deluca@opbg.net (M.D.L.); sara.chiurchiu@opbg.net (S.C.); fippolita.calo@opbg.net (F.I.C.C.); stefania.bernardi@opbg.net (S.B.); 4 Department of Clinical Sciences and Translational Medicine, University of Rome “Tor Vergata”, 00133 Rome, Italy; cifaldi@med.uniroma2.it (L.C.); bei@med.uniroma2.it (R.B.); 5 Fetal Medicine and Surgery Unit, Bambino Gesù Children’s Hospital, IRCCS, 00165 Rome, Italy; leonardo.caforio@opbg.net; 6 Department of Systems Medicine, University of Rome “Tor Vergata”, 00133 Rome, Italy

**Keywords:** NK cells, mother-to-child CMV transmission, congenital CMV infection, predictive biomarkers

## Abstract

Human cytomegalovirus (CMV) is a common herpesvirus causing lifelong latent infection in most people and is a primary cause of congenital infection worldwide. Given the role of NK cells in the materno-fetal barrier, we investigated peripheral blood NK cell behavior in the context of CMV infection acquired during pregnancy. We analyzed the NK phenotype and CD107a surface mobilization on PBMCs from CMV-transmitting and non-transmitting mothers and newborns with or without congenital infection. NK cells from non-transmitting mothers showed the typical phenotype of CMV-adaptive NK cells, characterized by higher levels of NKG2C, CD57, and KIRs, with reduced NKG2A, compared to transmitting ones. A significantly higher percentage of DNAM-1+, PD-1+, and KIR+NKG2A-CD57+PD-1+ CD56dim cells was found in the non-transmitting group. Accordingly, NK cells from congenital-CMV (cCMV)-infected newborns expressed higher levels of NKG2C and CD57, with reduced NKG2A, compared to non-congenital ones. Furthermore, they showed a significant expansion of CD56dim cells co-expressing NKG2C and CD57 or with a memory-like (KIR+NKG2A-CD57+NKG2C+) phenotype, as well as a significant reduction of the CD57-NKG2C- population. Degranulation assays showed a slightly higher CD107a geomean ratio in NK cells of mothers who were non-transmitting compared to those transmitting the virus. Our findings demonstrate that both CMV-transmitting mothers and cCMV newborns show a specific NK profile. These data can guide studies on predicting virus transmission from mothers and congenital infection in infants.

## 1. Introduction

Human cytomegalovirus (CMV) is a double-stranded DNA virus that belongs to the Herpesviridae family and is characterized by long-life persistence within the infected host. CMV can be extremely dangerous in vulnerable populations and can be responsible for severe congenital disease when it reaches the fetus through the placenta. In particular, congenital CMV (cCMV) infection is the most common congenital viral infection and among the main non-genetic causes of neurological pathologies, including psycho-motor retardation, cerebral palsy, and hearing loss [1,2,3]. About 10% of newborns infected during pregnancy show symptoms at birth, with a variable spectrum represented by low weight, petechiae, jaundice, hepatosplenomegaly, and microcephaly. Of these children, more than 90% will develop long-term sequelae and a percentage varying between 10 and 30% will not survive. Among asymptomatic newborns at birth, approximately 10–15% will show signs and symptoms later on, mainly hearing loss [4,5,6]. The transmission rate of the virus from mother to child varies depending on the gestational age of maternal infection (on average 5.5% or 21.0% during the preconception or periconception period, and 36.8%, 40.3%, or 66.2% in the first, second, or third trimester, respectively). The infection type also plays a role in transmission, with an average of 30–40% in primary infections and 1–2% in secondary infections, thus suggesting a role for maternal immunity in transmitting the virus to the fetus [7,8]. Natural killer (NK) cells belong to the innate branch of the immune system and account for 10–15% of circulating lymphocytes in human peripheral blood (PB), but are also resident in both lymphoid organs and several non-lymphoid tissues including the liver, lungs, gut, and uterus [9,10]. NK cells play an important role in the immune response against CMV as demonstrated by the multiple immune evasion strategies developed by the virus to escape their control [11,12,13]. Several studies have been conducted to define NK cell behavior during CMV infection. In particular, it has been reported that CMV can shape the repertoire/distribution of NK cell receptors, inducing the preferential expansion of a specific NK cell population (called memory-like or adaptive) co-expressing the activating receptor NKG2C and the terminal differentiation marker CD57. This CMV-induced NK cell subset displays a terminally differentiated phenotype and acquires some hallmarks of adaptive immunity (e.g., clonal expansion, increased longevity, enhanced effector functions, and epigenetic modifications). Adaptive NK cells display functional enhancement in terms of antibody-dependent cytotoxic activity and IFN-γ production [14,15,16,17,18,19,20]. Interestingly, CMV infection, along with other unknown co-factors, can induce an additional subset of fully mature NK cells characterized by the expression of the inhibitory receptor PD-1, not necessarily co-expressed with NKG2C [21]. The role of NK cells in the context of CMV infection during pregnancy is not yet clarified. During pregnancy, the mother is in a state of partial immunosuppression to prevent fetus rejection [22]. Uterine NK cells (called decidual NK, dNKs) are responsible for normal placental immunological homeostasis [23,24,25]. dNKs are CD56bright and compensate for poor cytotoxic capacity with the production of antiviral cytokines, such as IFN-γ, in the uterine microenvironment. Although dNKs typically express high levels of inhibitory receptors, including NKG2A, LIR-1, and KIRs, they undergo a receptor repertoire shift in response to CMV-infected fibroblasts towards activating receptors such as NKG2C, which allow them to carry out cytotoxic functions specifically directed against infected cells [14,26,27]. Regarding newborns, the literature evidence shows that particular NK cell subsets play a role in CMV infection. NKG2C+ NK cell expansion is particularly marked in infants with cCMV, especially if symptomatic, compared to asymptomatic and uninfected newborns. NKG2C+ NK cell increases seemed to be correlated with the viral load peak, thus implying that this cell population is involved in viremia control [28,29].

Based on this evidence, we characterized the phenotype and degranulation potential of PB NK cells isolated from mothers infected with CMV during pregnancy, their newborns, and children with cCMV infection, in order to understand NK cells’ role in CMV transmission. These data will contribute to refining predictive algorithms for congenital infections, potentially influencing the development of health policies.

## 2. Materials and Methods

### 2.1. Study Participants

**Figure 1:**
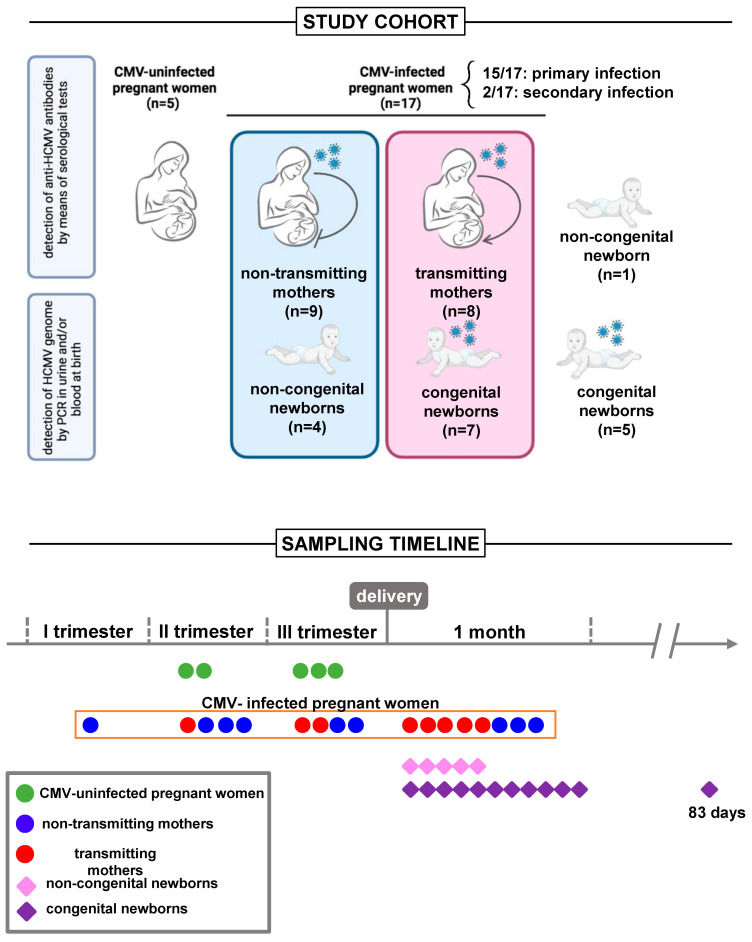
Study design. Study cohort: Seventeen CMV-infected pregnant women, with primary (n = 15) or secondary (n = 2) CMV infection, were enrolled in the study. Among the eleven mother/child dyads, seven women gave birth to children congenitally infected with CMV and four to children who did not experience CMV infection. Additional children, born to mothers with CMV infection during pregnancy, with (n = 5) or without (n = 1) cCMV, were included in the study, albeit in the absence of maternal sampling. A control group of CMV-uninfected pregnant women (n = 5) was also included. Sampling timeline: Blood sampling was performed on all uninfected pregnant women before delivery, two in the second, and three in the third trimester. Among CMV-infected pregnant women, eight were subjected to a postpartum blood draw simultaneously with their babies, nine before delivery, one in the first trimester, and four in the second and third trimesters. Sixteen newborns underwent blood sampling within the first month of life, and one child at 83 days of age.

Seventeen CMV-infected pregnant women were enrolled from June 2019 to June 2021 at Bambino Gesù Children’s Hospital in Rome, Italy, as part of the cohort study named PROTECT (Figure 1, study cohort). Only healthy women with uncomplicated pregnancies were included in our study. CMV infection was diagnosed by means of serological tests. Maternal anti-CMV IgG and IgM antibody detection was carried out by the clinical laboratory using standard diagnostic CLIA tests as previously described [30]. The diagnosis of primary CMV infection was made by (a) CMV seroconversion from negative to positive IgG during gestation, (b) the detection of IgG and IgM with low IgG avidity, or (c) the detection of IgG intermediate avidity in the presence of positive viral tests (CMV-qPCR on body fluids). The diagnosis of secondary infection was established in cases where a four-fold increase in anti-CMV IgG titer was observed, or when both IgG and IgM tested positive alongside high IgG avidity. Additionally, a positive result from CMV PCR in at least one sample from urine, saliva, or blood was required for confirmation.

Among the seventeen enrolled mothers, one woman opted for termination of the pregnancy following positive amniocentesis results indicating CMV detection in the amniotic fluid. Additionally, five mothers declined to grant consent for sampling their child, all of them making this decision after receiving a negative neonatal diagnosis for congenital CMV. Of the remaining eleven mother/child dyads, seven women gave birth to children congenitally infected with CMV and four mothers gave birth to children who did not experience CMV infection. Additional children, born to mothers with CMV infection during pregnancy, with (n = 5) or without (n = 1) cCMV were included in the study, albeit in the absence of maternal sampling. cCMV was defined by the qPCR detection of the CMV genome (DNA) in urine or blood within the first 3 weeks of life. Children with cCMV were divided into symptomatic and asymptomatic according to clinical manifestations [31]. Asymptomatic children were evaluated four times in the first year of life and two times in the second year. The follow-up included pediatric clinical evaluation, audiologic, ophthalmic, and neurodevelopmental assessment, and cerebral and abdominal imaging. Individualized follow-up was implemented for symptomatic patients based on the specific symptoms presented. The study also included a control group of healthy CMV-uninfected pregnant women (n = 5). These women underwent comprehensive blood evaluations encompassing testing for several pathogens along with hormone level assessments, facilitating the exclusion of pregnancy-related comorbidities that could impact study outcomes. None experienced any pregnancy complication. All paired study groups were age-matched. The study was approved by the local Research and Ethics Committee and written informed consent was obtained from all participants or legal guardians. 

### 2.2. Sample Collection and Storage

We analyzed maternal and infant peripheral blood samples, with blood sampling performed on all uninfected pregnant women before delivery, two in the second, and three in the third trimester. Regarding the CMV-infected pregnant women, eight of them were subjected to a postpartum blood draw simultaneously with their babies, whereas nine were subjected to a blood draw before delivery, one in the first trimester, and four in the second and third trimesters. Sixteen newborns underwent blood sampling within the first month of life, and one child at 83 days of age (Figure 1, sampling timeline). Venous blood was collected in EDTA tubes and processed within 2 h. Plasma was isolated from blood and stored at −80 °C. Peripheral blood mononuclear cells (PBMCs) were isolated from blood with a Ficoll density gradient and cryopreserved in fetal bovine serum (FBS), 10% dimethyl sulfoxide (DMSO), and liquid nitrogen until analysis.

### 2.3. NK Cell Immunophenotype

To evaluate, by multicolor flow cytometry, the expression of NKG2C, NKG2A, CD57, NKG2D, DNAM-1, KIRs, and PD-1 on NK cells, thawed PBMCs were stained with the LIVE/DEAD™ Fixable Near-IR Dead Cell stain dye (from Invitrogen, Waltham, MA, USA) for 15 min at room temperature (RT). Before fixing for 20 min at RT with 1% PFA, cells were stained for 20 min at 4 °C with PerCP-Cy5.5-conjugated anti-CD56, BV510-conjugated anti-CD16, Alexa Fluor 700-conjugated anti-CD3, BV605-conjugated anti-CD14, BV650-conjugated anti-CD19, PE-CF594-conjugated anti-NKG2D, BV786-conjugated anti-DNAM-1, BV421-conjugated anti-PD-1 (all from BD Biosciences, San Jose, CA, USA), PE-conjugated anti-NKG2C, FITC-conjugated anti-NKG2A (both from Miltenyi Biotec, Bergisch Gladbach, DE), PE-Cy7-conjugated anti-CD57 (from Life Technologies, Carlsbad, CA, USA), APC-conjugated anti-CD158 (KIR2DL1/S1/S3/S5), and anti-CD158b/j (KIR2DL2/L3) (both from BioLegend, San Diego, CA, USA) mAbs. Stained samples were acquired at CytoFLEX Flow Cytometer from Beckman Coulter Life Sciences and all results were analyzed using FlowJo version X.0.7 software.

### 2.4. NK Cell Degranulation Assays

To determine, by multicolor flow cytometry, CD107a (LAMP-1) surface mobilization on NK cells, thawed PBMCs were co-cultured (1:1) with human erythroleukemia K562 target cells for 4 h at 37 °C in the presence of FITC-conjugated anti-CD107a (from BD Pharmingen, San Diego, CA, USA) mAb. After the first hour, 0.66 μL/mL of BD GolgiStop (protein transport inhibitor containing monensin) was added. At the end of stimulation, cells were washed with PBS + 1% FCS + 5mM EDTA to promote conjugate disruption and stained with the LIVE/DEAD™ Fixable Near-IR Dead Cell stain dye (from Invitrogen, Waltham, MA, USA) for 15 min at RT. Before fixing for 20 min at RT with 1% PFA, cells were stained for 20 min at 4 °C with BV510-conjugated anti-CD16, PE-CF594-conjugated anti-CD3, BV605-conjugated anti-CD14, APC-conjugated anti-CD57, BV786-conjugated anti-DNAM-1, BV421-conjugated anti-PD-1 (all from BD Biosciences, San Jose, CA, USA), PerCP-conjugated anti-CD56 (from Invitrogen, Waltham, MA, USA), APC Alexa Fluor 750-conjugated anti-CD19 (from Life Technologies, Carlsbad, CA, USA), PE-conjugated anti-NKG2C (from Miltenyi Biotec, Bergisch Gladbach, DE, USA), and PE-Cy7-conjugated anti-NKp46 (from BioLegend, San Diego, CA, USA) mAbs. Stained samples were acquired using a CytoFLEX Flow Cytometer from Beckman Coulter Life Sciences and all results were analyzed using FlowJo version X.0.7 software.

### 2.5. Flow Cytometry Data Analysis (tSNE)

Data were analyzed with FlowJo software (version 10.8.1). All data depicted are gated on live cells, as determined by the LIVE/DEAD™ Fixable Near-IR Dead Cell stain dye. For visualizing high-dimensional flow cytometry data, we used the t-distributed stochastic nearest neighbor embedding (tSNE), a nonlinear dimensionality reduction algorithm. With tSNE, individual events are mapped onto a two-dimensional graph where data points representing cells with similar properties in high-dimensional space are grouped together in the reduced data space. The “DownSample” FlowJo plugin was run on NK cells in order to reduce and make uniform the population sizes for the further concatenation of samples from different groups into a single FCS file. tSNE was performed on the concatenated file using the “TSNE” plugin and the maps generated using data from the following compensated parameters as inputs: CD56, CD16, NKG2C, NKG2A, NKG2D, DNAM-1, CD57, KIR2DL1/S1/S3/S5, KIR2DL2/L3, and PD-1 for the phenotype analysis and CD56, CD16, NKG2C, DNAM-1, CD57, NKp46, PD-1, and CD107a for the degranulation analysis; under the following tSNE settings: iteration 1000, perplexity 30, learning rate (Eta) 910–2100, the ANNOY algorithm as the k nearest neighbors (KNN) algorithm and the fast Fourier transform (FTT) interpolation as the gradient algorithm, resulting in tSNE plots with less than 5 million events (Figure S1).

### 2.6. Statistical Analysis

Statistical analyses were performed using Graph Pad Prism 8 (Graph Pad Software, Inc., San Diego, CA). The D’Agostino–Pearson test was used to assess data distribution and statistical comparisons, with the t-test being performed for normally distributed data, or with the non-parametric unpaired (Mann–Whitney) test. All tests were two-tailed and statistical significance was set at *p* < 0.05. The Supplementary table reports all the study results.

## 3. Results

### 3.1. Clinical Laboratory Characterization

From June 2019 to June 2021, we enrolled seventeen mothers (mean age 32.31 ± 4.076 SD) with primary (n = 15) or secondary (n = 2) CMV infection, their newborns (n = 11, 7 congenitally infected with CMV and 4 who did not experience CMV infection), and children with (n = 5) or without (n = 1) cCMV, albeit in the absence of maternal sampling (Figure 1). Table 1 describes the general and routine laboratory characteristics of congenital and non-congenital children. Primary infection during pregnancy was predominant in both child groups. All babies were born at term (37–41 weeks of gestational age), with the exception of two congenital neonates born at 35 and 35 weeks + 6 days, respectively. Table 2 shows the clinical characteristics of congenital children (n = 12). Among these, around 25.0% of cases (3/12) showed symptoms at birth. Of these, two children exhibited unilateral sensorineural hearing loss. Similarly, magnetic resonance imaging (MRI) showed alterations in around 25.0% of cases (3/12). Four children (33.3%) showed abnormalities on a brain ultrasound. Among the congenital newborns, six (50.0%) were treated with Valganciclovir for 6 months, according to infectious disease specialist prescriptions. The treatment was initiated whenever the infection was classified as symptomatic at birth (retinal, auditory, CNS abnormalities, etc.) as defined by one of the most recent consensus conferences on the topic [32]. The treatment started within 3 months of age. A blood draw was always performed before the start of treatment, except in two cases sampled 3 and 7 days after, respectively. Although it is reported that Valganciclovir treatment could induce changes in markers of immune cells [33], our results were not greatly affected by removing the two newborns from the analysis. To date, the follow-up average duration of infected children is 24.18 months. Periodic checks showed the onset of sequelae in 58.3% of cases (7/12) (Table 2). 

### 3.2. The Maturation Profile of NK Cells Is Influenced by CMV Infection and Exhibits Variations among Study Cohorts

**Figure 2:**
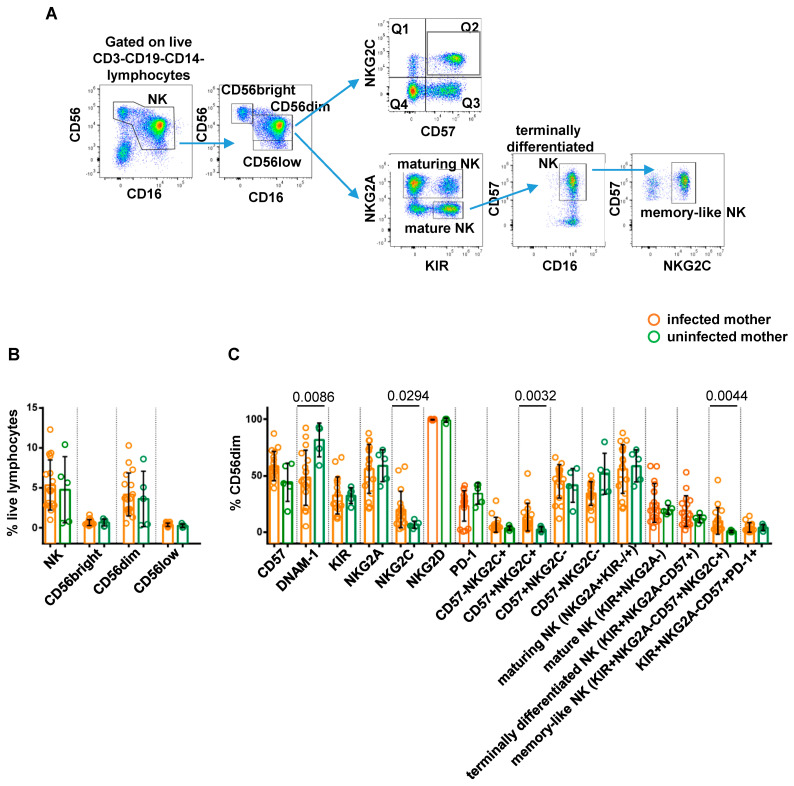
Comparative analysis of NK cell profile in CMV-infected vs. uninfected pregnant women. Representative gating strategy for CMV-induced memory-like NK cell subsets is shown in panel (**A**). Scatter plots with bar (mean ± SD) depict frequency distribution with respect to (**B**) live lymphocytes of NK (CD56+CD16+/−), CD56bright (CD56brightCD16−), CD56dim (CD56dimCD16+), and CD56low (CD56lowCD16+) cells gated on CD3-CD19-CD14− cells. (**C**) CD56dim cells of subpopulations distinguished on the basis of surface expression levels of CD57, DNAM-1, KIR, NKG2A, NKG2C, NKG2D, and PD-1 markers, either alone or in combination. Mann–Whitney test was used to assess differences in cell frequencies between CMV-infected (orange dots, n = 17) and uninfected (green dots, n = 5) pregnant women. Significance was set at *p* < 0.05.

**Figure 3:**
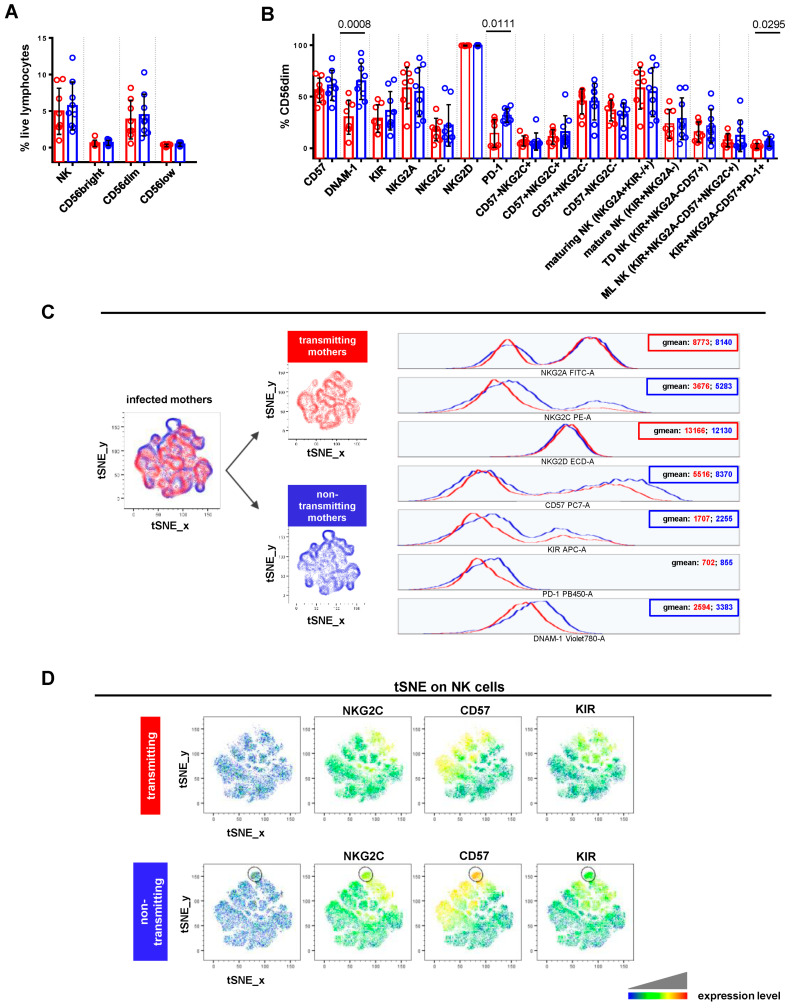
Comparative analysis of NK cell profile in CMV-transmitting vs. non-transmitting mothers. (**A,B**) Scatter plots with bar (mean ± SD) depict frequency distribution, as in Figure 2B,C. Unpaired t test and Mann–Whitney test were used to assess differences in cell frequencies between transmitting (red dots, n = 8) and non-transmitting (blue dots, n = 9) mothers. Significance was set at *p* < 0.05. (**C**) tSNE algorithm was configured to distribute data combined from transmitting and non-transmitting mothers’ samples according to the expression of NK cell markers CD56, CD16, NKG2C, NKG2A, CD57, NKG2D, DNAM-1, KIRs, and PD-1. Multigraph histogram overlays with geomean values were generated in a combined FCS file obtained by concatenating 2000 events in NK cell down-sample (identified as CD3-CD19-CD14- live lymphocytes) of transmitting (red, n = 7) and non-transmitting (blue, n = 8) mothers. (**D**) By using the same combined FCS file, single parameter heatmaps were obtained for transmitting (upper panels) and non-transmitting (lower panels) groups. Outline population (black circle) is specific to non-transmitting mothers and only the expressed markers are reported. TD NK: terminally differentiated NK; ML NK: memory-like NK.

**Figure 4:**
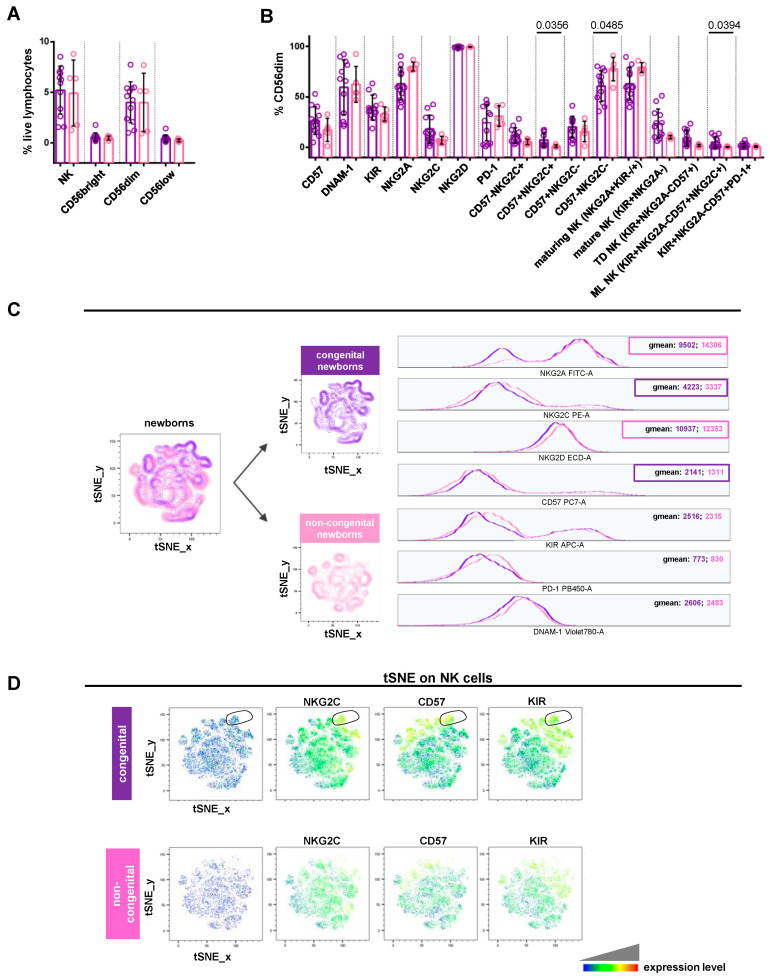
Comparative analysis of NK cell profile in newborns with or without CMV congenital infection. (**A**,**B**) Scatter plots with bar (mean ± SD) depict frequency distribution, as in Figure 2B,C. Mann–Whitney test was used to assess differences in cell frequencies between congenital (purple dots, n = 12) and non-congenital (pink dots, n = 5) newborns. Significance was set at *p* < 0.05. (**C**) tSNE algorithm was configured to distribute data combined from congenital and non-congenital children’s samples according to Figure 3C. Multigraph histogram overlays with geomean values were generated in a combined FCS file obtained by concatenating 1880 events in NK cell down-sample of congenital (purple, n = 11) and non-congenital (pink, n = 5) children. (**D**) By using the same combined FCS file, single parameter heatmaps were obtained for congenital (upper panels) and non-congenital (lower panels) groups. Outline population (black circle) is specific to congenital children and only the expressed markers are reported. TD NK: terminally differentiated NK; ML NK: memory-like NK.

Figure 2A depicts the applied gating strategy for NK phenotype analysis. No significant differences in the frequency of total NK (identified as CD3-CD19-CD14-CD56+ live lymphocytes), CD56brightCD16−, CD56dimCD16+, and CD56lowCD16+ cells were observed between the paired groups (Figure 2B, Figure 3A and Figure 4A). We next evaluated the differential expression of NK cell markers in each study cohort. When comparing CMV-infected and uninfected mothers, a significantly higher frequency of NKG2C+ (*p* = 0.0294), NKG2C+CD57+ (*p* = 0.0032), and memory-like (defined as KIR+NKG2A-CD57+NKG2C+; *p* = 0.0044) CD56dimCD16+ cells was observed in the infected group, consistent with previous findings [34,35,36], while intriguingly, the size of DNAM-1+ CD56dimCD16+ cells was significantly lower in the same group of infected mothers (*p* = 0.0086; Figure 2C). Overall, these results indicated that NK cells from CMV-infected mothers in our cohort exhibited a phenotype typical of adaptive NK cells associated with CMV infection [18,37]. 

Focusing on CMV-infected pregnant women, non-transmitting mothers displayed a significantly higher frequency of DNAM-1+ (*p* = 0.0008), PD-1+ (*p* = 0.0111), and KIR+NKG2A-CD57+PD-1+ (*p* = 0.0295) CD56dimCD16+ cells, compared to transmitting ones (Figure 3B). A similar trend, though not statistically significant, was also observed for CD56dimCD16+ cells expressing KIR (mean 36.27 ± 18.72 SD vs. 28.71 ± 13.45), CD57 (mean 60.98 ± 14.47 SD vs. 56.36 ± 11.75), and NKG2C (mean 22.14 ± 20.21 SD vs. 17.99 ± 10.95), alone or in combination (mean 15.72 ± 15.77 SD vs. 10.94 ± 6.95). Furthermore, non-transmitting mothers showed a slightly increased frequency of mature (mean 28.59 ± 20.29 SD vs. 23.52 ± 13.85), terminally differentiated (mean 21.56 ± 16.35 SD vs. 15.79 ± 9.69), and memory-like (mean 12.07 ± 15.27 SD vs. 7.70 ± 6.40) CD56dimCD16+ populations. As shown in Figure 3C, NK cells from the non-transmitting group expressed higher levels of NKG2C (geomean: 5283 vs. 3676), CD57 (geomean: 8370 vs. 5516), KIRs (geomean: 2255 vs. 1707), and DNAM-1 (geomean: 3383 vs. 2594). This phenotype was found on CD56dimCD16+, CD56bright CD16-, and CD56lowCD16+ cells (Figure S2A). Instead, NK cells from non-transmitting mothers exhibited lower levels of NKG2A (geomean: 8140 vs. 8773) and NKG2D (geomean: 12,130 vs. 13,166) compared to transmitting ones (Figure 3C). Thanks to tSNE plots generated for NK cells, we could identify one population, almost exclusively exhibited by non-transmitting mothers, characterized by a high expression of CD57 and, to a lesser extent, NKG2C and KIRs (Figure 3D, outlined population). These results indicated that NK cells from non-transmitting mothers showed a more typical phenotype of CMV-adaptive NK cells than transmitting ones. 

When comparing congenital and non-congenital newborns, we observed a significantly higher frequency of NKG2C+CD57+ (*p* = 0.0356) and memory-like (*p* = 0.0394) CD56dimCD16+ cells, as well as a significant (*p* = 0.0485) reduction in the CD57-NKG2C- population in the congenital group (Figure 4B). In the same group, a greater frequency, though not statistically significant, was observed for CD56dimCD16+ expressing CD57 (mean 26.52 ± 13.68 SD vs. 17.27 ± 11.36), KIRs (mean 39.62 ± 12.31 SD vs. 33.12 ± 6.925), and NKG2C (mean 18.09 ± 13.77 SD vs. 7.206 ± 3.784), or exhibiting a mature (mean 23.62 ± 14.58 SD vs. 10.07 ± 1.805), terminally differentiated (mean 9.315 ± 7.785 SD vs. 1.940 ± 1.146), or KIR+NKG2A-CD57+PD-1+ (mean 1.978 ± 2.267 SD vs. 0.81 ± 0.6702) phenotype (Figure 4B). As shown in Figure 4C, NK cells from congenital newborns expressed higher levels of NKG2C (geomean: 4223 vs. 3337) and CD57 (geomean: 2141 vs. 1311). This phenotype was found on CD56dimCD16+, CD56brightCD16-, and CD56lowCD16+ cells (Figure S2B). By contrast, NK cells from the congenital group exhibited lower levels of NKG2A (geomean: 9502 vs. 14,306) and NKG2D (geomean: 10,937 vs. 12,353) compared to the non-congenital one (Figure 4C). tSNE plots generated for NK cells identified one population typical of congenital newborns expressing high levels of NKG2C, CD57, and KIRs (Figure 4D, outlined population). These results showed that NK cell phenotypes from congenital newborns recapitulate the CMV-adaptive NK cell scenario, similar to that found in non-transmitting mothers. Overall, this is in line with the previously well-documented expansion of memory-like or adaptive NK cells in older children and CMV-infected adults [34,35,36].

### 3.3. Degranulating NK Cells Show a Different Phenotype among Study Cohorts

**Figure 5:**
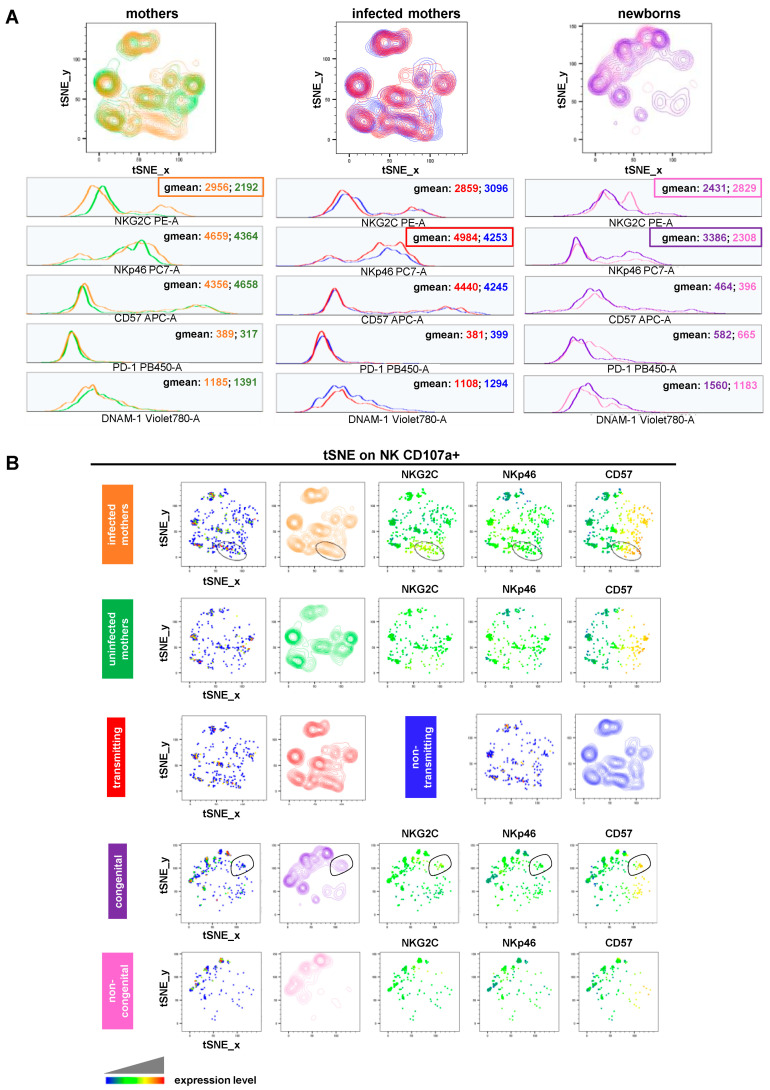
NK cell degranulation (**A**) tSNE algorithm was generated for CD107a+ NK cells and configured to distribute data combined from study cohorts of stimulated samples according to the expression of NK cell markers CD56, CD16, CD107a, NKG2C, CD57, NKp46, DNAM-1, and PD-1. Multigraph histogram overlays with geomean values were generated in two combined FCS files. One was obtained by concatenating 1000 events in NK cell down-sample of either CMV-infected (orange, n = 9) and uninfected (green, n = 4) pregnant women or transmitting (red, n = 5) and non-transmitting (blue, n = 4) mothers. The other one was obtained by concatenating 1180 events in the NK cell down-sample of congenital (purple, n = 7) and non-congenital (pink, n = 5) children. (**B**) By using the same combined FCS files, single parameter heatmaps were obtained for each study group. Outline populations (black circles) are specific to infected pregnant women and congenital children, and only the expressed markers are reported.

Finally, we carried out assays of NK cell degranulation (evaluated as CD107a surface mobilization) by stimulating donor-derived PBMCs with the human erythroleukemia K562 target cells (Figure S3A). While no statistically significant differences were identified among the study cohorts, noteworthy observations were made. We observed a slightly higher CD107a geomean ratio (stimulated/unstimulated sample) in the total NK cells of CMV-uninfected (green, mean value ± SD: 1405 ± 0.1482) compared to infected (orange, mean value ± SD: 1275 ± 0.1390) pregnant women (Figure S3B). This is in line with evidence from the literature describing how “memory” NK cells that expanded following CMV infection are significantly different from “conventional” ones due to their functional capabilities [37,38,39] showing, among others, a poorer reactivity toward tumor targets in terms of both degranulation and IFN-γ production [37,40,41]. A similar trend for the CD107a geomean ratio was also found in the total NK cells of mothers who were non-transmitting (blue, mean value ± SD: 1302 ± 0.1008) compared to those transmitting (red, mean value ± SD: 1252 ± 0.1698) the virus (Figure S2B). Importantly, we then compared the phenotype of degranulating (CD107a+) NK cells across the study cohorts. We observed that, in comparison to uninfected pregnant women, infected ones exhibited activated NK cells expressing elevated levels of NKG2C (geomean: 2956 vs. 2192) (Figure 5A, left panels). A comparative analysis of non-transmitting vs. transmitting mothers revealed a different NK cell profile: lower levels of NKp46 (geomean: 4253 vs. 4984) (Figure 5A, middle panels). Conversely, degranulating NK cells of congenital newborns showed more NKp46 (geomean: 3386 vs. 2308) but less NKG2C (geomean: 2431 vs. 2829) than those of non-congenital ones (Figure 5A, right panels). Looking at the tSNE plots generated for CD107a+ NK cells, we identified a single population, which is typical of infected but not uninfected pregnant women, and that was positive for CD57, NKG2C, and to a lesser extent, NKp46 (Figure 5B, upper panels). Similarly, a single population expressing CD57, NKG2C, and to a lesser extent, NKp46 was also found to be specific to congenital newborns (Figure 5B, lower panels).

## 4. Discussion

Patient enrollment started in 2019. The unforeseen challenges posed by the COVID-19 pandemic, coupled with the resultant mobility restrictions, resulted in a lower enrollment rate than initially expected. Despite these challenges, exacerbated by the non-recommendation of routine serologic screening for CMV infection during pregnancy, the successful enrollment of seventeen pregnant women represents a significant milestone. Although this cohort offers a valuable opportunity to gather preliminary insights, another limitation is represented by the variable sampling time in pregnant mothers. As reported by Wisgalla A et al., in healthy pregnancy peripheral blood, NK cells showed an increase in CD56bright and a decrease in CD56dim populations toward the third trimester [42]. In our cohort, CD56dim population frequency was not affected by third trimester sampling. Fifteen pregnant women experienced a primary infection and two a secondary one. Seven newborns were positive for congenital CMV infection. The transmission rates found in our cohort (42.8%, 33.3%, and 75% in the first, second, and third trimester, respectively) were relatively in line with epidemiological data reported in the literature [7]. Considering the placenta lymphocyte composition and the maternal–fetal transmission mechanism of CMV [23,24,25], our study focused on assessing the behavior of PB NK cell subsets in mothers and their babies, in order to understand their role in CMV transmission. NK cells constitute the predominant population of lymphocytes at the maternal–fetal interface in the uterus, representing 70% of the resident immune cells and ensuring immune homeostasis thanks to their marked proliferative and immune regulatory ability through the secretion of various cytokines and chemokines [23]. Vertical CMV transmission is thought to result, in most cases, from placenta infection via maternal circulation. It has been hypothesized that CMV is transmitted from infected maternal uterine to decidual cells, where it would replicate and from which it would spread to the placenta, causing cytotrophoblast focal infection. Maidji E et al. demonstrated than in women with a weak antibody response to CMV, IgG/virion immune complexes were subjected to transcytosis via the neonatal FcR expressed by placental syncytiotrophoblasts, thus allowing the virus to avoid being killed by chorionic villi macrophages and to disseminate to fetal vessels [43,44,45,46,47]. The main objective of this study was to define immunological markers predictive of the maternal–fetal CMV infection transmission in mothers who acquired or reactivated CMV infection during pregnancy. The investigated markers allowed us to characterize NK cell subsets in different stages of differentiation as well as to identify NK cells with different functional capabilities. The comprehensive immunophenotypic study unveiled distinct NK cell behaviors across the study cohorts. Most results are consistent with what is expected in response to CMV infection: shaping NK cell repertoire/distribution and fostering the expansion of fully mature NK cell populations, often referred to as memory or adaptive NK cells. These cells exhibit characteristics indicative of immunological memory, such as antigen-specific clonal-like expansions, inheritable cell-intrinsic modifications, an extended half-life, and heightened recall responses [18,21]. Indeed, the different NK cell characteristics found by the immunophenotypic study reflect NK cell activation/differentiation under viral pressure and are likely due to the immune response that women and newborns infected with CMV have developed to counteract the virus. In particular, a higher frequency of CD56dimCD16+ populations preferentially expressing KIRs, the activating receptor NKG2C, and the terminal maturation/senescence marker CD57, alone or in combination, was observed in CMV-infected compared to uninfected women/newborns. In line with these data, tSNE plots generated for NK cells identified a population almost exclusively exhibited by congenital newborns that expressed high levels of NKG2C, CD57, and KIRs. Notably, the NK cell phenotype found in CMV-infected women/newborns was more mature (KIR+NKG2A-), including terminally differentiated (KIR+NKG2A-CD57+) and memory-like (KIR+NKG2A-CD57+NKG2C+) subsets, compared to uninfected individuals. These cells, after encountering the virus, start a process of maturation and differentiation to fight the infection more effectively. Conversely, the NK cell phenotype found in the CMV-uninfected women/newborns included less mature subsets or NK cells in a “resting” state. This was demonstrated by the expression of receptors associated with the early phases of NK cell development (e.g., inhibitory marker NKG2A) as well as by the lack of both NKG2C and CD57 expression. In addition, the size of DNAM-1+CD56dimCD16+ cells was significantly lower in CMV-infected pregnant women compared to uninfected ones. Of note, DNAM-1 is known to be a costimulatory molecule essential for the optimal differentiation of memory NK cells, from a mouse model of CMV-infection [48], and a crucial activating receptor recognizing ligands expressed in response to CMV-infection [49]. Therefore, after CMV infection, the expression of the DNAM-1 receptor appeared down-regulated, thus leading us to hypothesize that, as an evasion strategy, viral proteins can negatively modulate the expression of DNAM-1 in addition to its ligands [49,50]. Among infected pregnant women, non-transmitting mothers exhibited a greater NK cell activity as witnessed by a more mature/senescent immunophenotypic profile than transmitting ones. Looking at tSNE plots generated for NK cells, we identified one population typical of non-transmitting mothers that expressed high levels of CD57 and, to a lesser extent, NKG2C and KIRs. By contrast, in transmitting mothers, NK cells did not reach activation and senescent levels comparable to those not transmitting the virus. Notably, non-transmitting mothers displayed a significantly higher frequency of DNAM-1+CD56dimCD16+ cells, thus supporting the role played by this receptor in maternal immunity against CMV infection [51,52]. It is well-known from the literature that “memory” NK cells that expanded following CMV infection are significantly different from “conventional” ones due to their functional capabilities [37,38,39] showing, among others, a poorer reactivity toward tumor targets in terms of both degranulation and IFN-γ production [37,40,41]. In line with this, we observed a slightly higher CD107a geomean ratio in the total NK cells of CMV-uninfected compared to infected pregnant women. A similar trend was also found in the NK cells of non-transmitting mothers compared to those transmitting. This finding led us to hypothesize that in addition to previously described non-neutralizing Fc-mediated antibody effector functions (e.g., IgG activation of FcγRI/FcγRII and antibody-dependent cellular phagocytosis/ADCP, as well as FcγRIIIA/CD16 and antibody-dependent cellular cytotoxicity/ADCC activation) [53,54], direct cytotoxic killing by NK cells may constitute a component of protective maternal immunity against cCMV that is potentially associated with a reduced risk of vertical transmission. tSNE plots generated for degranulating (CD107a+) NK cells enabled the identification of a single population, which is typical of infected but not uninfected pregnant women, and that was positive for CD57, NKG2C, and to a lesser extent, NKp46. Similarly, a single population expressing CD57, NKG2C, and to a lesser extent, NKp46, was also found to be specific to congenital newborns. Recently, several groups demonstrated the expression of PD-1, a marker previously considered exclusively pertinent to T cells, on NK cells in response to specific stimuli [21]. Our data highlighted a difference in PD-1 expression between CMV-infected mothers transmitting the virus and those not transmitting it. Indeed, non-transmitting mothers exhibited a greater frequency of PD-1+ on both CD56dim CD16+ and terminally differentiated NK cells; in summary, non-transmitting mothers showed more active and “exhausted” NK cells that had completely ended their potential of antagonizing the virus. Therefore, PD-1 expression on fully mature NK cells may be an index of both NK cell exhaustion and the subsequent regulatory mechanisms that are activated to limit an excessive NK cell response and damages resulting from their hyperactivity against the virus. In conclusion, these data support an NK-mediated immune response more vigorous and effective in infected pregnant women that is able to prevent the maternal–fetal transmission of CMV infection. Further analysis of secondary infection with respect to primary infection during pregnancy should be performed on a larger cohort size to fully establish the impact of viral infection on the NK cell maturation profile, which is likely driven by chronic antigen stimulation.

## Figures and Tables

**Table 1:** General and routine laboratory characteristics of congenital and non-congenital children study groups. HC, head circumference; WBC, white blood cells; PLT, platelet; Hb, hemoglobin; GOT, glutamic–oxaloacetic transaminase; GPT, glutamic pyruvic transaminases; CRP, C reactive protein; ns, not significant.

	Neonates with cCMV (n = 12)	Neonates without cCMV (n = 5)	*p*-Value
sex males/females	7/5	3/2	ns
maternal infection type primary/secondary	10/2	3/2	0.0022
trimester of maternal infection I/II/III	4/1/6 *	3/1/1	-
term/pre-term birth (37–41 wk)	10/2	5/0	0.0346
small-for-gestational-age (SGA) neonates	0/12	0/5	-
APGAR score (9–10)	12/0	5/0	-
weight at birth, gr (mean ± SD)	3110 ± 580.3	3023 ± 40.41	ns
HC at birth, cm (mean ± SD)	33.85 ± 2.001	34.63 ± 0.75	ns
WBC, μL (mean ± SD)	11,479 ± 2578	8990 ± 2464	ns
lymphocytes, μL (mean ± SD)	7500 ± 1917	5850 ± 1437	ns
neutrophils, μL (mean ± SD)	1988 ± 796.7	1645 ± 766.2	ns
PLT, μL (mean ± SD)	369,083 ± 136,276	505,750 ± 139,636	ns
monocytes, μL (mean ± SD)	907.5 ± 200.7	830 ± 332.2	ns
Hb, g/dL (mean ± SD)	12.41 ± 2.057	13.63 ± 3.827	ns
GOT, UI/L (mean ± SD)	54.45 ± 21.80	43.40 ± 13.76	ns
GPT, UI/L (mean ± SD)	36.82 ± 19.80	26.80 ± 11.88	ns
CRP, mg/dL (mean ± SD)	0.1025 ± 0.1664	0.046 ± 0.02074	ns

* data not available for a pregnant woman.

**Table 2:** Clinical characteristics of congenital children study cohort. MRI, magnetic resonance imaging.

Congenital Children (n = 12)	n (%)
symptoms *	3 (25.0%)
MRI *	3 (25.0%)
brain ultrasound *	4 (33.3%)
abdominal ultrasound *	neg
audiological examination *	2 (16.7%)
fundus examination *	neg
other symptoms *	4 (33.3%)
therapy, Valganciclovir	6 (50.0%)
follow-up average duration, months	24.18
sequelae at follow-up	7 (58.3%)
expressive language delay	6/7
attention difficulties	2/7
sensorineural hearing loss	2/7
epileptic encephalopathy	1/7
motor retardation	2/7
spastic tetraparesis	1/7
delay in psychomotor skills	1/7
delay in oral feeding skills	1/7

* at birth.

## Data Availability

The data that support the findings of this study are available from the corresponding author, [DA], upon reasonable request.

## Supplementary Materials

The following supporting information can be downloaded at: https://www.mdpi.com/article/10.3390/v16050780/s1, Figure S1: tSNE workflow for NK cell phenotypic and degranulation analysis; Supplementary table; Figure S2: Phenotypic profile of CD56bright, CD56dim and CD56low cell populations; Figure S3: CD107a surface mobilization on NK cells.
