# Supplementary material for: Characterization of Natural Killer Cell Profile in a Cohort of Infected Pregnant Women and Their Babies and Its Relation to CMV Transmission
Source: Viruses. 2024 May 14;16(5):780. doi: 10.3390/v16050780 (PMC11125694; doi:10.3390/v16050780)
Supplement: Supplementary file 1 [file viruses-16-00780-s001.zip › viruses-2958831-supplementary.pdf]

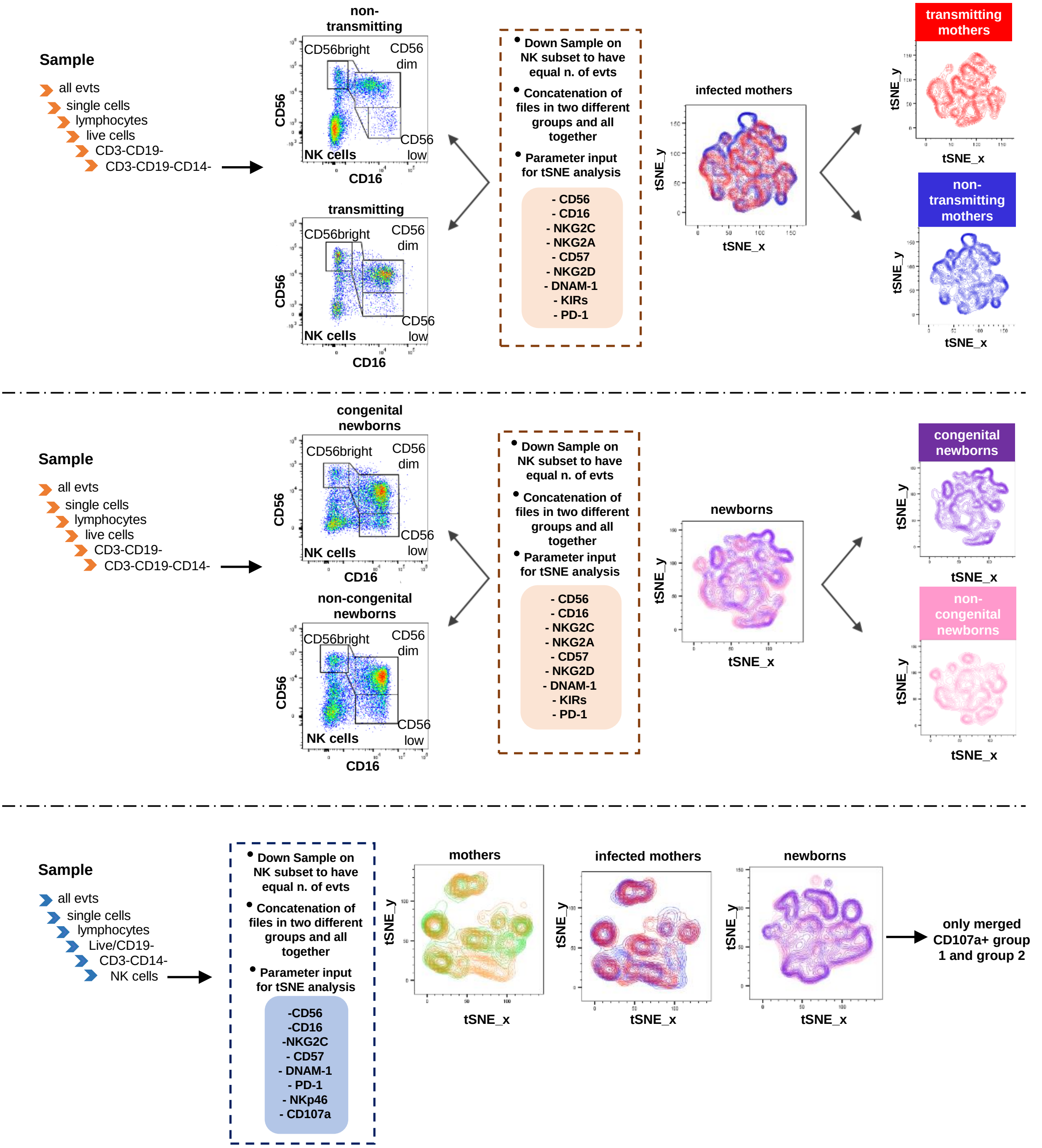

Supplementary Figure S1. tSNE workflow for NK cell phenotype and degranulation. tSNE was performed on the concatenated file using the “TSNE” plugin, being the maps generated using data from the following compensated parameters as input: CD56, CD16, NKG2C, NKG2A, NKG2D, DNAM-1, CD57, KIR2DL1/S1/S3/S5, KIR2DL2/L3 and PD-1 for the phenotype analysis and CD56, CD16, NKG2C, DNAM-1, CD57, NKp46, PD-1 and CD107a for the degranulation analysis.

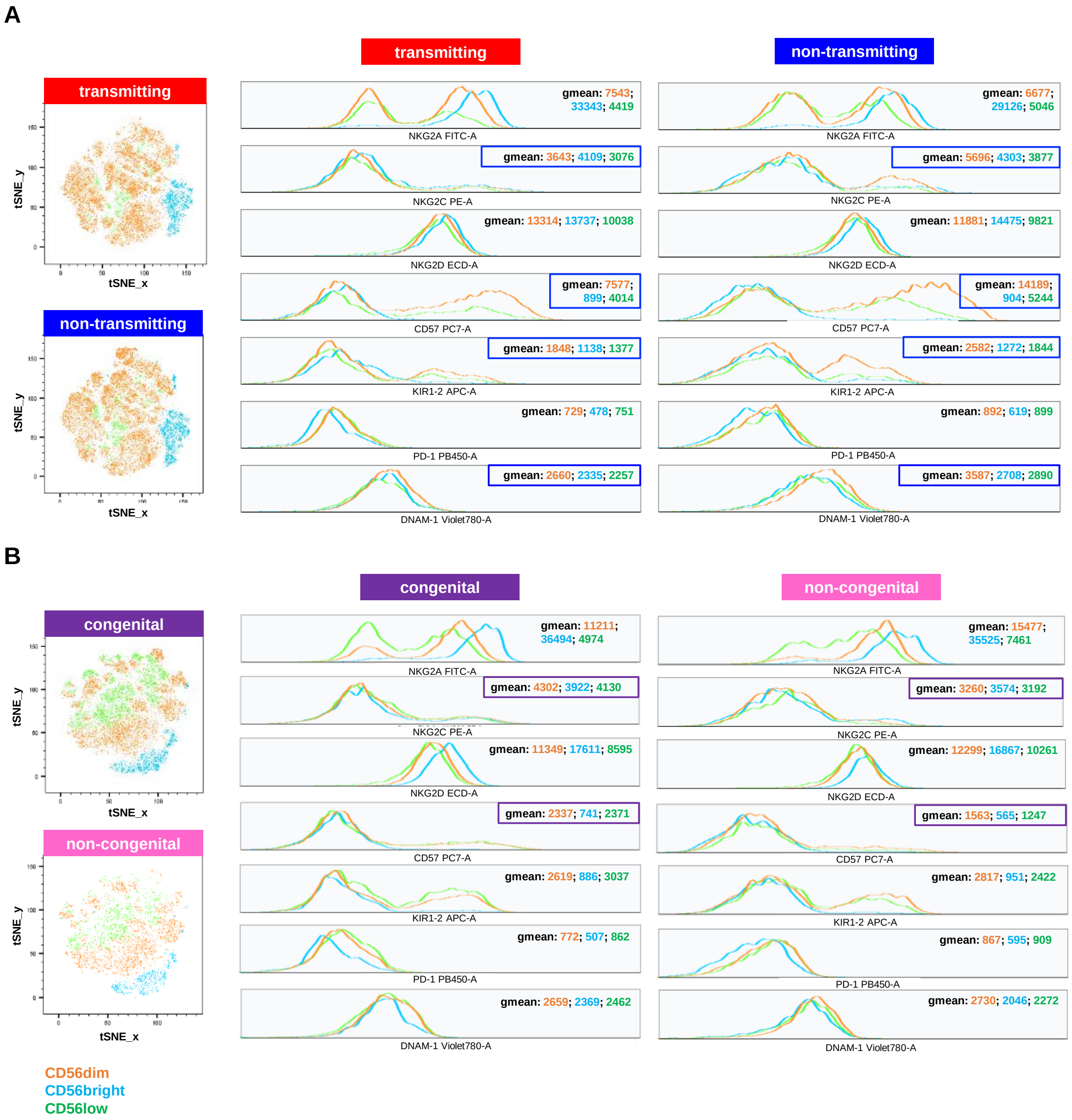

Supplementary Figure S2. Phenotypic profile of CD56bright, CD56dim and CD56low cell populations. Multigraph histogram overlays with geomean values for CD56bright (blue), CD56dim (orange) and CD56low (green) cells were generated with a combined FCS file obtained by concatenating (**A**) 2000 events in NK cell DownSample of transmitting (n=7, left panels) and non-transmitting (n=8, right panels) mothers or (**B**) 1880 events in NK cell DownSample of congenital (n=11, left panels) and non-congenital (n=5, right panels) newborns.

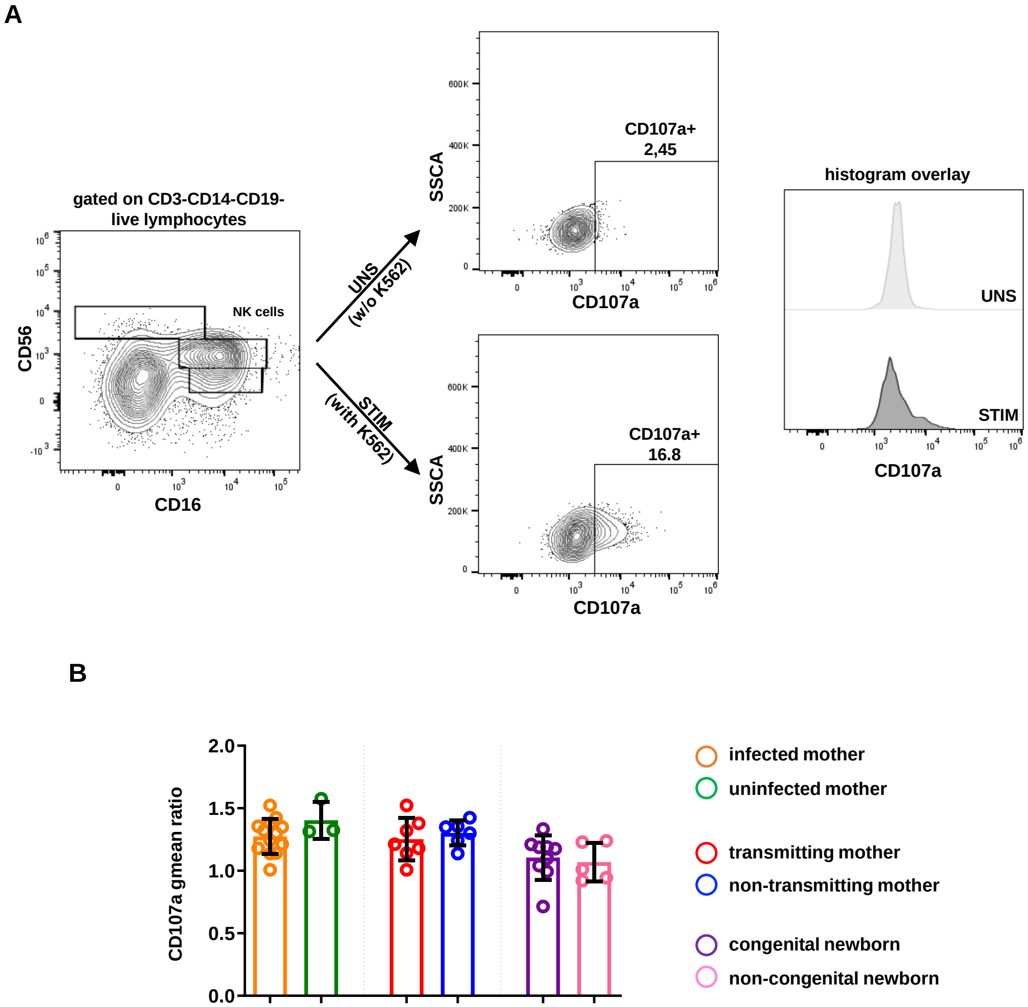

Supplementary Figure S3. CD107a surface mobilization on NK cells. Representative gating strategy for NK cell degranulation is shown in **panel A**. (**B**) Scatter plots with bar (mean ± SD) depict CD107a geomean ratio (stimulated/not stimulated) of CMV-infected (orange dots, n=13) and –uninfected (green dots, n=3) pregnant women or transmitting (red dots, n=7) and non-transmitting (blue dots, n=6) mothers or congenital (purple dots, n=9) and non-congenital (pink dots, n=5) newborns. Mann Whitney test was used to assess differences between study groups. Significance was set at $p < 0.05$.
